# Genome-wide profiling of the microRNA-mRNA regulatory network in skeletal muscle with aging

**DOI:** 10.18632/aging.100677

**Published:** 2014-07-12

**Authors:** Ji Young Kim, Young-Kyu Park, Kwang-Pyo Lee, Seung-Min Lee, Tae-Wook Kang, Hee-Jin Kim, So Hee Dho, Seon-Young Kim, Ki-Sun Kwon

**Affiliations:** ^1^Aging Research Institute, Korea Research Institute of Bioscience & BioTechnology, 125 Gwahak-ro, Yuseong-gu, Daejeon, Korea; ^2^Medical Genomics Research Center, Korea Research Institute of Bioscience & BioTechnology, 125 Gwahak-ro, Yuseong-gu, Daejeon, Korea; ^3^Department of Functional Genomics, University of Science and Technology, Daejeon, Korea

**Keywords:** Skeletal muscle, aging, microRNA profiling, imprinted Dlk1-Dio3, microRNA cluster, novel microRNA

## Abstract

Skeletal muscle degenerates progressively, losing mass (sarcopenia) over time, which leads to reduced physical ability and often results in secondary diseases such as diabetes and obesity. The regulation of gene expression by microRNAs is a key event in muscle development and disease. To understand genome-wide changes in microRNAs and mRNAs during muscle aging, we sequenced microRNAs and mRNAs from mouse gastrocnemius muscles at two different ages (6 and 24 months). Thirty-four microRNAs (15 up-regulated and 19 down-regulated) were differentially expressed with age, including the microRNAs miR-206 and -434, which were differentially expressed in aged muscle in previous studies. Interestingly, eight microRNAs in a microRNA cluster at the imprinted *Dlk1-Dio3* locus on chromosome 12 were coordinately down-regulated. In addition, sixteen novel microRNAs were identified. Integrative analysis of microRNA and mRNA expression revealed that microRNAs may contribute to muscle aging through the positive regulation of transcription, metabolic processes, and kinase activity. Many of the age-related microRNAs have been implicated in human muscular diseases. We suggest that genome-wide microRNA profiling will expand our knowledge of microRNA function in the muscle aging process.

## INTRODUCTION

The progressive loss of skeletal muscle mass, strength, and quality, also known as sarcopenia, is a common characteristic of aging [1]. Decreased muscle mass and function accompany reduced physical activity and energy expenditure in the elderly. Consequently, sarcopenia results in muscle weakness, leading to disability and metabolic disorders, including insulin resistance, type 2 diabetes, and hypertension. Although, multiple factors contributing to sarcopenia are characterized by using transcriptome and proteome approaches [2], little is known about the molecular mechanisms of aging-related changes in skeletal muscle.

MicroRNAs(miRNAs) are endogenous, noncoding, short (20–22 nucleotides) RNAs that are involved in biological processes such as metabolism, development, cancer, and aging [3]. A long primary miRNA is initially transcribed by RNA polymerase II and processed in the nucleus by the endonuclease Drosha into precursor miRNA (pre-miRNA). The pre-miRNA is then exported into the cytoplasm and further cleaved by Dicer to generate mature miRNAs. MiRNAs recognize 3' untranslated regions, they are guided by RNA inducing silencing complex (RISC), and they silence the target's expression by either translational inhibition or degradation. The binding of miRNA to a target gene depends on the seed sequence (2–8 nucleotides) with imperfect base pairing. Therefore, a single miRNA can regulate multiple mRNAs, and vice versa, a single mRNA can be targeted by multiple miRNAs. MiRNA genes are distributed across diverse genomic locations; some are isolated, but approximately 50% of miRNAs are found in clusters transcribed as polycistronic miRNA transcripts [4].

Recently, several works have demonstrated that various miRNAs are differentially expressed during aging and participate in the aging processes in tissue- and cell-type specific manners [5, 6]. For example, in the liver, age-regulated miRNAs such as miR-34, -93, and -214 target a gene important for oxidative stress defense and decrease its activity with aging [7]. In the brain, nearly 39% of age-regulated miRNAs are predicted to target components that play a critical role in oxidative phosphorylation [8]. A few groups have reported the profiling of miRNA expression in skeletal muscle with aging using microarray analysis [9, 10]. Let-7 family members were elevated in aged human skeletal muscle, contributing to reduced cellular proliferation and regenerative capacity in aged human muscle [9, 11]. Aging altered the expression of 57 miRNAs in mouse quadriceps muscles and it was suggested that age-regulated miRNAs decreased proliferation and favored the terminal differentiation of myogenic precursor [10]. Furthermore, resistance exercise, caloric restriction, or nutrient-related hormones such as the adipokine leptin changed the expression of age-regulated miRNAs, suggesting that these outputs may reverse muscle atrophy or induce changes to a younger phenotype [12, 13]. In addition to the normal muscle aging process, miRNAs participate in muscle pathologies such as muscular dystrophy and rhabdomyosarcoma. A total of 185 known miRNAs are deregulated in 10 major muscular disorders in humans and, of these deregulated miRNAs, five miRNAs, miR-146b, -221, -222, -155, and -214, were consistently regulated in almost all samples [14]. In rhabdomyosarcoma, which is a type of cancer originating from skeletal muscle progenitors, miR-1/206 and -29 directly targeted c-Met and YY1, respectively, suggesting a potential tumor suppressor role [15, 16].

Here, we profiled both miRNA and mRNA expression in young and aged mouse gastrocnemius muscles to investigate the potential role of miRNAs in skeletal muscle aging through next-generation sequencing (NGS) technology, which permits the precise identification of mature and novel miRNAs [17]. We found 34 age-related miRNAs in aged muscle and found that 57% of the down-regulated miRNAs are enriched as a cluster in the *Dlk1-Dio3* imprinted genomic region on mouse distal chromosome 12. In combination with mRNA expression, we found that the genes targeted by age-related miRNAs in skeletal muscle appear to contribute to muscle aging by regulating transcription.

## RESULTS

### miRNA profiling using next-generation sequencing (NGS) in skeletal muscle

To study aging in skeletal muscle, we compared the muscle mass from young mice (6-month-old) to that of aged mice (24-month-old). Aged mice showed a significant loss of muscle mass in both the tibialis anterior (TA) and the gastrocnemius muscle (Fig. S1A). We focused particularly on the gastrocnemius muscle, which is a large skeletal muscle that gradually declines in mass through the age of 32 months (Fig. S1B).

**Figure 1:**
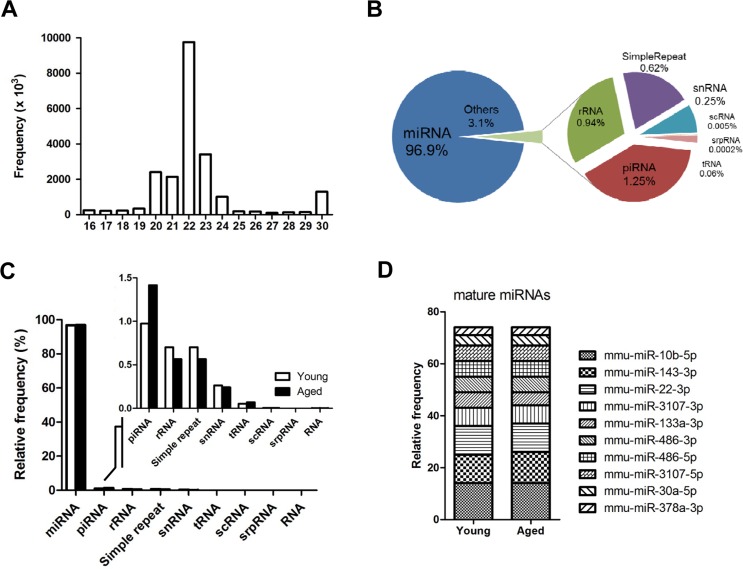
Overview of next-generation sequencing of the small RNA transcriptome in skeletal muscle (**A**) Size distribution of the preprocessed small RNA reads after trimming the adaptor sequence. Reads < 16 nucleotides were discarded, and reads > 30 nucleotide were shortened to 30 nucleotides on the X-axis. The Y-axis depicts their frequencies. A peak for miRNA candidates (22 nucleotide) is centered. (**B**) Portions of the small RNA types in the total preprocessed reads based on their mapped locations referring to the gene annotation from the UCSC Genome database. The proportion of miRNA is approximately 97%, and the other 3% is made up of other small RNAs and repeat types including rRNA, piRNA, snRNA, scRNA, srpRNA, tRNA, and simple repeats. (**C**) Composition of different types of small RNAs in the preprocessed reads based on the gene annotation from the UCSC Genome database. This graph shows the same information as panel B in Figure 1, but the read contents for young and aged mice are distinguished. (**D**) The ten most abundant mature miRNAs constitute ~75% of the total miRNA reads in skeletal muscle. The proportion of the miRNAs was based on the mapped read population in the preprocessed read set of the young mouse samples referring to the annotated genomic locations of the miRNA genes by the UCSC Genome Browser (GRCm38/mm10). The relative frequency of each miRNA to the total miRNA was calculated by dividing the individual miRNA reads by the total number of miRNA reads.

To understand the molecular mechanisms of muscle aging, we analyzed the genome-wide miRNA expression of young and aged skeletal muscles by small-RNA sequencing. As shown in figure 1A, most sequences were 20~23 nucleotides in length with a peak at 22 nucleotides, representing mature miRNAs (96.9%). The remaining small RNAs (3.1%) included snRNA, scRNA, srpRNA, rRNA, tRNA, and piRNA (Fig. 1B). Interestingly, piRNA, mainly identified in germ cells, was up-regulated with aging (Fig. 1C, inset graph). The piwi-piRNA mechanism has a role in maintaining genome integrity and telomere protection in *Drosophila* [18], which might be a novel modulator of muscle aging and longevity. To see the abundance of mature miRNAs in skeletal muscle, we identified the top 10 miRNAs in the sequence reads. In skeletal muscle, the 10 most highly expressed mature miRNAs comprised nearly 75% of the total miRNAs, showing a relatively even distribution in abundance (Fig. 1D). Many of the top 10 miRNAs were previously reported for their roles in muscle. For example, two well-known miRNAs, miR-133 and miR-486, modulate skeletal muscle proliferation and differentiation [19]. MiR-143 regulates smooth muscle fate by promoting differentiation and repressing the proliferation of smooth muscle cells [20]. Recently, miR-22 was identified as a cardiac- and skeletal muscle-enriched miRNA that is up-regulated during myocyte differentiation and cardiomyocyte hypertrophy [21]. MiR-30a promotes muscle differentiation by inhibiting Snai1/2 in primary myoblast [22]. Finally, myogenic factor MyoD negatively regulates myogenic repressor MyoR via miR-378 during myoblast differentiation [23]. However, for the most abundant miR-10b, with over 6,000,000 reads per million (RPM), there were no previous reports concerning its role in either aging or muscle development.

### Differentially expressed miRNAs in aged skeletal muscle

High throughput small-RNA sequencing detected 603 and 703 mature miRNAs having at least 5 reads from young and aged skeletal muscles, respectively. Among them, 20 miRNAs were up-regulated and 19miRNAs were down-regulated in aged muscle (>1.5-fold difference, *t*-test, *P*<0.05) (Fig. S2). The selected miRNAs included several miRNAs that were previously reported to be involved in muscle aging. For example, miR-206 was up-regulated in aged human skeletal muscle, while miR-434 was down-regulated in aged mouse skeletal muscle [10, 12]. MiR-34, which is up-regulated with aging and modulates neurodegeneration in *Drosophila* and in aged heart by regulating cardiac aging [24], was also increased in aged skeletal muscle.

**Figure 2:**
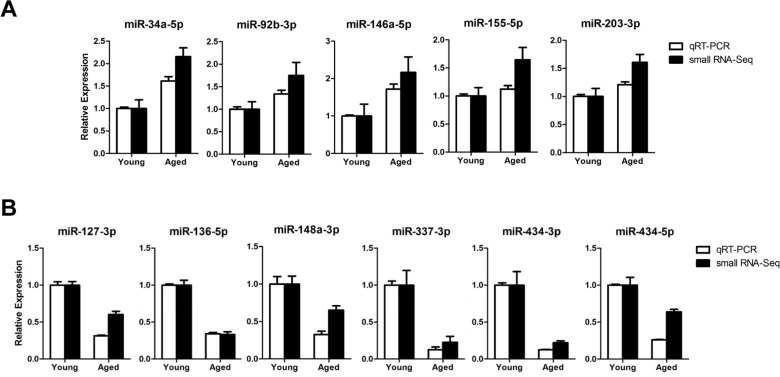
Expression confirmation of 11 representative miRNAs by qRT-PCR (**A**-**B**) Relative expressions of miRNAs were determined by TaqMan qRT-PCR (white bar, *n* = 4 for each group) or small RNA-sequencing (black bar, *n* = 6 for each group) of the gastrocnemius muscle. The qRT-PCR results were normalized by the average of the U6 snRNA and relative expression level of aged muscles compared to that of young muscles was shown. Error bars indicate SEM.

To validate the age-related miRNA identified by sequencing, we analyzed 11 representative miRNAs by TaqMan quantitative real-time PCR (qRT-PCR) on gastrocnemius muscles from young (6-month-old) or aged (24-month-old) mice. The qRT-PCR results for down-regulated miRNAs were consistent with the results of the sequencing (Fig. 2B). All five up-regulated miRNAs from sequencing data was consistently increased in qRT-PCR, though a modest up-regulation was observed in two miRNAs (Fig. 2A). To exclude ambiguity, we chose two cut-offs, >2 for up-regulation and >1.5 for down-regulation, and we selected 15 up-regulated and 19 down-regulated miRNAs (Tables 1 and 2).

**Table 1:** Up-regulated miRNAs in aged muscle MiRNAs with >1.5-fold expression changes between young (6-month-old) and aged (24-month-old) muscles are listed from top to bottom according to fold changes and values of differences given in log_2_. MiRNAs in bold indicate TaqMan qRT-PCR validation.

miRNA	Fold Change	*P*-value
mmu-miR-185-3p	5.1	0.0012
mmu-miR-615-5p	4.5	0.0128
mmu-miR-196b-3p	4.4	0.0179
mmu-miR-215-5p	4.3	0.0126
mmu-miR-3084-3p	4.2	0.0446
mmu-miR-6406	4.2	0.0193
mmu-miR-223-5p	3.9	0.0091
mmu-miR-483-3p	3.8	0.0077
mmu-miR-369-5p	3.6	0.0138
mmu-miR-7075-3p	3.3	0.0253
mmu-miR-7083-5p	2.9	0.0469
mmu-miR-206-3p	1.7	0.0216
mmu-miR-34c-5p	1.5	0.0110
**mmu-miR-34a-5p**	1.3	0.0275
**mmu-miR-146a-5p**	1.3	0.0325
mmu-miR-29b-3p	1.0	0.0218
mmu-miR-677-5p	0.9	0.0056
**mmu-miR-92b-3p**	0.8	0.0417
**mmu-miR-155-5p**	0.8	0.0392
**mmu-miR-203-3p**	0.8	0.0399

**Table 2:** Down-regulated miRNAs in aged muscle MiRNAs with >1.5-fold change in expression between young (6-month-old) and aged (24-month-old) muscles are listed from top to bottom according to fold changes and values of differences. MiRNAs in bold indicate TaqMan qRT-PCR validation.

miRNA	Fold Change	*P*-value
mmu-miR-337-5p	−5.2	0.0149
mmu-miR-3544-3p	−5.1	0.0147
mmu-miR-540-5p	−4.9	0.0200
**mmu-miR-337-3p*^a^***	−3.0	0.0324
mmu-miR-3544-5p*^a^*	−3.0	0.0308
**mmu-miR-434-3p**	−2.1	0.0001
mmu-miR-3071-5p	−2.0	0.0004
mmu-miR-136-3p*^a^*	−2.0	0.0004
mmu-miR-3071-3p*^a^*	−1.6	0.0000
**mmu-miR-136-5p**	−1.6	0.0000
mmu-miR-143-5p	−1.2	0.0004
mmu-miR-190a-5p	−1.0	0.0139
mmu-miR-872-3p	−0.9	0.0152
mmu-miR-193a-3p	−0.9	0.0164
mmu-miR-144-3p	−0.8	0.0298
**mmu-miR-127-3p**	−0.7	0.0002
**mmu-miR-434-5p*^a^***	−0.6	0.0082
**mmu-miR-148a-3p**	−0.6	0.0130
mmu-miR-411-5p	−0.6	0.0091

a miRNA* (passenger) strand processed from opposite arm of the mature miRNA.

### Down-regulation of miRNA cluster in aged skeletal muscle

There is some evidence that miRNAs act as a group or pack in the aging process [25]. Recently, it was reported that age-regulated miRNAs identified in long-lived individuals were enriched on four chromosomes, 7, 9, 17, and X [26]. In addition, 11 miRNA clusters have been shown to be affected in the aged heart [27]. To see whether the expression of miRNAs was affected individually or collectively during muscle aging, we examined the genomic locations of age-regulated miRNAs in skeletal muscle. Notably, 8 of the 14 down-regulated miRNAs (57%)were located in the imprinted *Dlk1-Dio3* genomic region on distal mouse chromosome 12 (see details in Discussion). Among them, seven miRNAs were distributed broadly across *Rtl1* and the small nucleolar RNA *Rian* within the *Dlk1-Dio3* region (Fig. S3). In contrast, the up-regulated miRNAs were randomly distributed throughout the genome.

### Novel miRNAs identified in skeletal muscle

**Figure 3:**
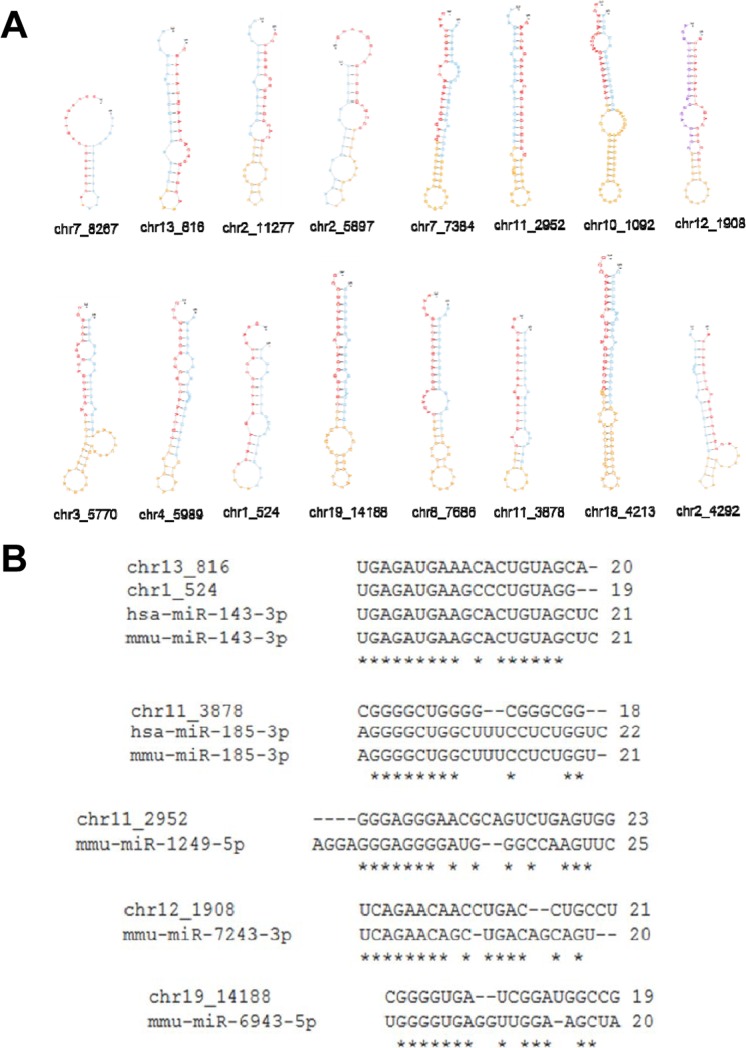
Sixteen novel miRNAs identified in skeletal muscle (**A**) Secondary structure of the hairpin according to 16 novel miRNAs detected in skeletal muscle. Red denotes a mature sequence. Yellow denotes a loop and blue denotes a star sequence. (**B**) Conservation of six novel miRNAs to known miRNAs. Asterisk indicates conserved sequence.

We used the miRDeep2 algorithm to identify novel miRNAs from our sequencing data [28]. Initially, miRDeep2 predicted 93 novel miRNAs. We then evaluated the candidates according to two criteria: 1) No mapping to sno/miRNA or non-coding RNAs according to the UCSC annotation. 2) Expression of at least two reads either in young or aged muscles. By these two criteria, the 93 candidates were narrowed down to 16 novel miRNAs (Table S1). The 16 novel miRNA precursor sequences folded well into a hairpin structure characteristic of *bona fide* miRNA (Fig. 3A). Six novel miRNAs shared homology with known miRNAs in their seed sequence (6–8 nucleotide long) as determined by the miRDeep2 algorithm, indicating that they may be new family members of those miRNAs (Fig. 3B). Interestingly, the mature sequences of two novel miRNAs (chr1_524 and chr13_816) were nearly identical to miR-143-3p, which regulates the fate of smooth muscle by promoting the differentiation and repressing the proliferation of smooth muscle cells [20]. Another novel miRNA, chr11_3738, shared homology with miR-185, which participates in 22q11DS and schizophrenia by dysregulating sarco/endoplasmic reticulum Ca^2+^-ATPase (SERCA2) [29]. While 2 of the novel miRNAs (chr7_8267 and chr13_816) exhibited high expression (>1,000 read counts), the remaining 14 novel miRNAs exhibited relatively low expression (<300 read counts). All of the novel miRNAs identified in skeletal muscle were distributed randomly in the genome.

### RNA sequencing analysis in skeletal muscle

**Figure 4:**
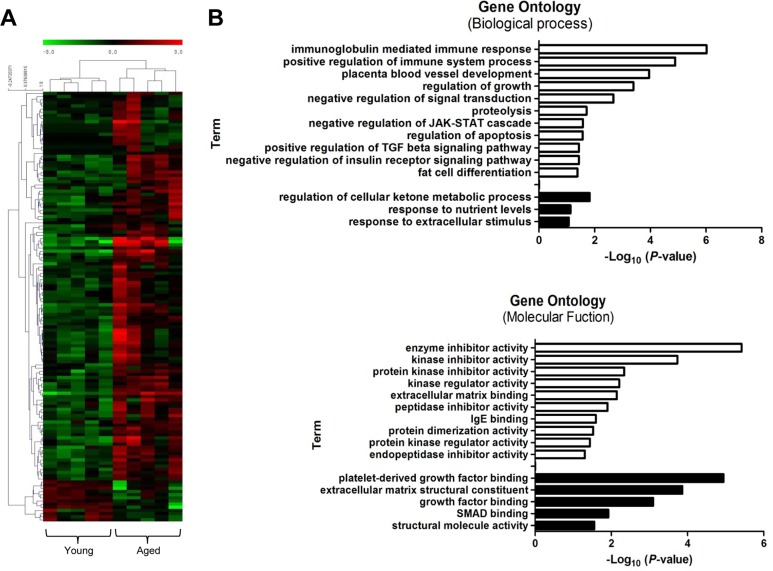
Transcriptional signature of aged skeletal muscle and its selected gene ontology terms of the mRNA transcriptome (**A**) Unsupervised hierarchical clustering of the 136 differentially regulated mRNAs with aging; 123 genes were up-regulated, and 13 genes were down-regulated. Each column presents the mRNA expression in young (*n* = 5) and aged (*n* = 5) muscle. The intensity represents the magnitude of the difference. Red and green denote high and low expression, respectively. (**B**) Bar plot for the -log_10_ of the *P*-value of the selected GO terms. The up- and down-regulated genes (2-fold change) were subjected to gene ontology analysis using DAVID. The enriched biological processes (upper) and molecular functions (lower) were plotted. White bar, enriched GO terms regulated by up-regulated genes; black bar, enriched GO terms regulated by down-regulated genes.

We performed RNA sequencing (RNA-Seq) to identify genes that were differentially expressed between young and old muscles. Overall, 123 genes were up-regulated, while only 13 genes were down-regulated, showing a dramatic increase in gene expression with muscle aging (>2-fold difference, *t*-test, *P*<0.05) (Fig. 4A). Young and aged samples were clearly separated into two distinct clusters by hierarchical clustering. Interestingly, 5 of the 123 up-regulated genes (4%) were over-expressed more than 7-fold. In contrast, all 13 down-regulated genes changed moderately. Several genes identified in RNA-Seq were previously reported to be associated with age-related diseases. Early growth response 1 (Egr1) is a transcriptional regulator that is also known as a major regulator of cell senescence [30]. Another identified gene, apolipoprotein D (Apod), has a role in the aging brain and in Alzheimer's dementia [31].

Next, we performed gene ontology (GO) analysis to understand the functional significance of the genes with changed expression. For the up-regulated genes, biological processes related to the immune response were highly over-represented: e.g., ISG15-protein conjugation, phagocytosis, engulfment, and complement activation (Fig. 4B, white bar). Other significantly enriched biological processes included the positive regulation of the transforming growth factor (TGF) beta signaling pathway and the negative regulation of the insulin pathway, apoptosis, and fat cell differentiation. Among these examples, the TGF beta pathway is a well-known regulator of skeletal muscle aging that represses myogenic differentiation and muscle regeneration [11]. In the ‘Molecular Functions’ category, up-regulated genes were significantly enriched for GO terms related to various enzyme activities such as kinase, peptidase, and endopeptidase. IgE binding, which is a molecular function related to the immune system and matrix binding, was also represented. The down-regulated genes were enriched in terms such as metabolic process, response to nutrient levels, platelet-derived growth factor binding, and SMAD binding (black bar).

### Putative target genes regulated by age-related miRNAs and their roles in biological processes

**Figure 5:**
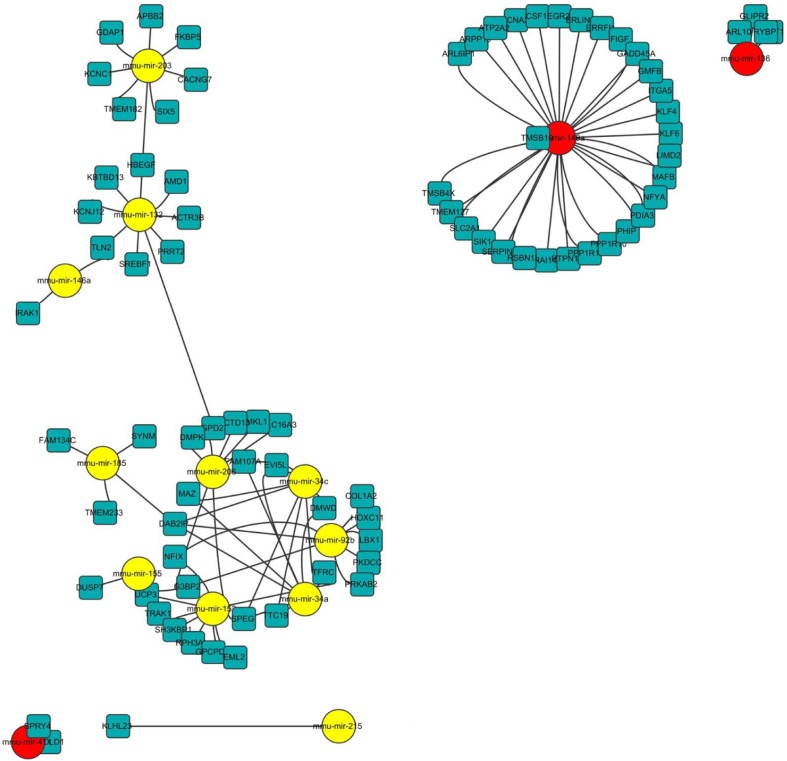
Network of miRNA-mRNA interaction The network displays the predicted interactions between age-related miRNAs and mRNAs from the sequencing and was generated using Cytoscape (version 3.0, www.cytoscape.org/). The up-regulated miRNAs are shown in yellow, down-regulated miRNAs in red. The miRNAs are depicted by circles and the mRNAs as squares. Their interaction is represented by one edge.

**Figure 6:**
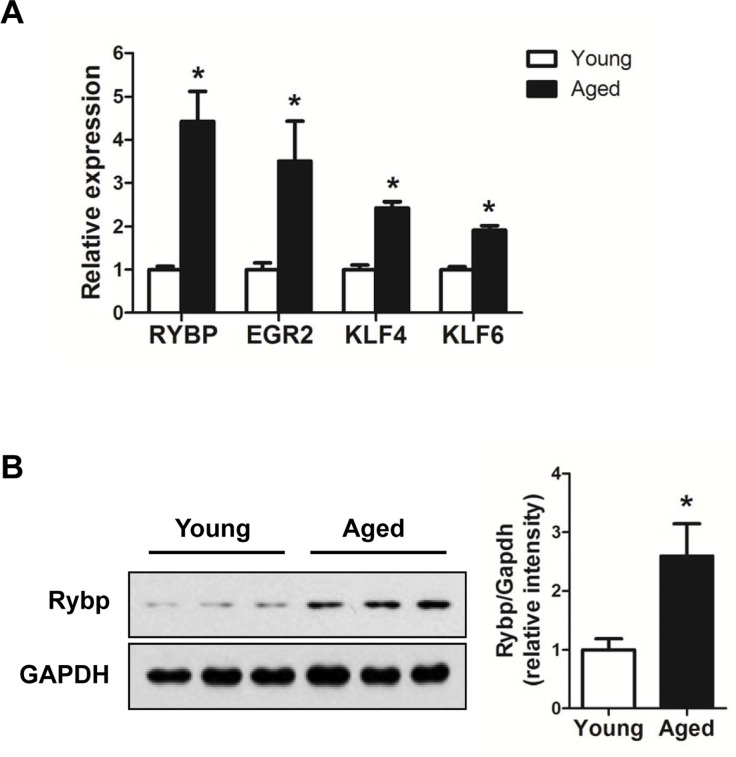
Up-regulation of genes involved in the trans-cription regulation in aged muscle (**A**) Relative expressions of 4 genes, Egr2, Klf4, Klf6, and Rybp, were determined by qRT-PCR (*n* = 3 for each group). The data were normalized to the amount of GAPDH mRNA. Error bars indicate SEM (Student's *t*-test, * *P* < 0.05). (**B**) Western blot and quantification of Rybp in young (white bar) and aged (black bar) gastrocnemius muscle (*n* = 3 for each group). GAPDH was used as loading control. * *P* < 0.05.

Next, we tried to understand the biological significance of age-regulated miRNAs by identifying their target mRNAs. We used the TargetScan program (version 6.2) to predict the biological targets of each miRNA and then selected genes that showed a negative correlation between mRNA (1.5-fold difference) and miRNA expression. A total of 94 mRNA-miRNA interactions were identified, including 37 up-regulated and 57 down-regulated genes targeted by 14 age-related miRNAs (Table 3 and Fig. 5). By gene ontology analysis, the genes targeted by down-regulated miRNAs represented two clusters with enrichment scores ≥1.3 (Table S2). Of note, we found that the most enriched term in ‘biological process’ category was the regulation of transcription, in which Egr2, Klf4, Klf6, and Rybp was involved. Consistent with our RNA-Seq data, we confirmed that mRNA levels of all four genes were increased in aged muscle by qRT-PCR (Fig. 6A). Among them, Rybp, Ring1 and YY1-binding protein, is known to negatively regulate skeletal myogenesis in injury-induced muscle regeneration [32]. We confirmed that Rybp was also increased at the protein level in aged muscle (Fig. 6B). These results suggested that Rybp and its putative regulatory miRNA, miR-136, could contribute to muscle aging process.

**Table 3:** De-regulated miRNAs and their target genes from RNA-Seq mRNAs are predicted as targets of up- or down-regulated miRNAs. TargetScan-predicted miRNA-mRNA pairs showing negative correlation in expression profile were selected.

Down-regulated miRNAs	Up-regulated target genes
mmu-mir-148a	ARL6IP1, ARPP19, ATP2A2, CCNA2, CSF1, EGR2, ERLIN1, ERRFI1, FIGF, GADD45A, GMFB, ITGA5, KLF4, KLF6, LIMD2, MAFB, NFYA, PDIA3, PHIP, PPP1R10, PPP1R12A, PTPN14, RAI14, RSBN1L, SERPINE1, SIK1, SLC2A1, TMEM127, TMSB10, TMSB4X
mmu-mir-411	APOLD1, SPRY4
mmu-mir-136	RYBP, ARL10, GLIPR2, UGGT1

Up-regulated miRNAs	Down-regulated target genes
mmu-mir-34a/c	DAB2IP, DMWD, EVI5L, FAM107A, MAZ, SPEG, TFRC, TTC19
mmu-mir-92b	COL1A2, DAB2IP, G3BP2, HOXC11, LBX1, NFIX, PKDCC, PRKAB2
mmu-mir-132	ACTR3B, AMD1, GPD2, HBEGF, KBTBD13, KCNJ12, PRRT2, SREBF1, TLN2
mmu-mir-146a	IRAK1, TLN2
mmu-mir-152	EML2, GPCPD1, NFIX, RPH3AL, SH3KBP1, TFRC, TRAK1, UCP3
mmu-mir-155	DUSP7, G3BP2
mmu-mir-185	DAB2IP, FAM134C, SYNM, TMEM233
mmu-mir-203	APBB2, CACNG7, FKBP5, GDAP1, HBEGF, KCNC1, SIX5, TMEM182
mmu-mir-206	DMPK, G3BP2, GPD2, KCTD13, MKL1, SLC16A3, SPEG
mmu-mir-215	KLHL23

### Disease enrichment analysis of age-related miRNAs

**Figure 7:**
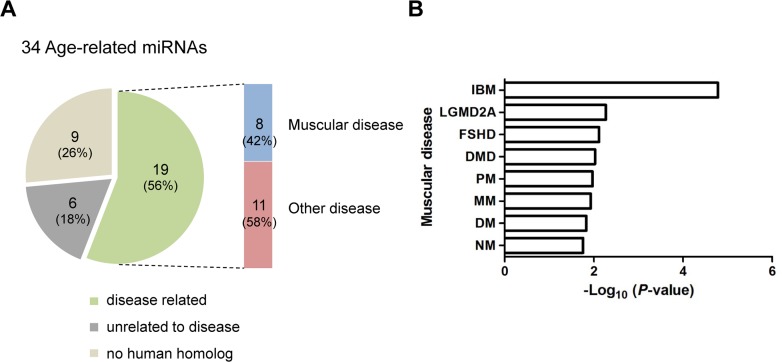
Association of age-related miRNAs identified in skeletal muscle with human diseases (**A**) The human homologues of 19 of 34 age-related miRNAs (56%) were associated with diseases. Among these, 8 miRNAs (42%) were related to human muscular diseases. Nine of the 34 miRNAs (26%) were not related to disease and 6 miRNAs (18%) had no human homologues. (**B**) Bar plot for the -log10 of the P-value of the 8 miRNAs enriched for muscular diseases. Becker muscular dystrophy, BMD; dermatomyositis, DM; Duchenne muscular dystrophy, DMD; facioscapulohumeral muscular dystrophy, FSHD; inclusion body myositis, IBM; limb-girdle muscular dystrophies types 2A, LGMD2A; Miyoshi myopathy, MM; nemaline myopathy, NM; polymyositis, PM; rhabdomyosarcoma, RMS.

MiRNAs are implicated in various diseases [3]. To examine whether the 34 age-related miRNAs identified in skeletal muscle are implicated in human disease, we used a human miRNA-associated disease database (HMDD), which provides information on changes in miRNA regulation in various human diseases [33]. As a result, human homologues corresponding to the 11 up-regulated and 8 down-regulated miRNAs were related to 58 and 40 human diseases, respectively (data not shown). Interestingly, 8 of the 19 miRNAs were related to human muscular diseases (Tables S3 and S4 and Fig. 7A). Notably, the changes in the miRNA expression level in those diseases were correlated with up-regulation rather than down-regulation. For example, the human homologues for five miRNAs that were up-regulated in aged muscle were also up-regulated in muscular diseases, except for miR-29b in DMD and RMS. In contrast, only one miRNA, hsa-miR-193, was down-regulated in DMD. In addition, 61 miRNAs related to 10 different groups of human muscle disorders are annotated in HMDD. We found that 8 miRNAs were significantly enriched in 8 muscular diseases, such as Duchenne muscular dystrophy (DMD), facioscapulohumeral muscular dystrophy (FSHD), limb-girdle muscular dystrophies types 2A (LGMD2A) (Fig. 7B). This analysis thus represents a meaningful relationship between the age-regulated miRNAs identified in mouse skeletal muscle and human muscular diseases.

## DISCUSSION

Significant advances have been made regarding the roles of miRNAs in aging processes through massive analyses of miRNAs that are differentially expressed with aging in various tissues. Here, we systematically profiled miRNA and mRNA gene expression from young and aged skeletal muscle. A total of 34 age-related miRNAs were identified. Of the down-regulated miRNAs, miR-148a-3p and -127-3p belonged to the 50 most abundant miRNAs in skeletal muscle (Fig. S4, Asterisk). MiR-127 was identified as a cardiac valve-enriched miRNA [34]; however, there were no previous reports on its role in skeletal muscle development. MiR-148a, one of the down-regulated miRNAs, was identified as a myogenic miRNA that promotes myogenic differentiation through the ROCK1 gene. MiR-155, one of the up-regulated miRNAs, represses skeletal muscle differentiation by repressing MEF2A [35]. Thus, in accordance with their expression and role in myogenesis, miR-148a and miR-155 might be involved in the aging process through muscle differentiation or regeneration.

Several groups have reported genome-wide profiling of miRNA expression in skeletal muscle with aging. The expression of 57 miRNAs in mouse quadriceps muscles was altered by aging using a miRNA array [10]. In another study, 18 age-associated miRNAs were identified in aged human skeletal muscles by miRNA array [9]. Recently, age-associated miRNA alterations in the skeletal muscle were reported in rhesus monkeys based on small RNA sequencing [13]. The differentially expressed miRNAs in previous studies appeared consistently in mouse skeletal muscle from our study; for example, up-regulated miRNAs such as miR-29b, -34a, -206, -215, and -223 in aged muscle were also elevated, and the down-regulated miRNA miR-434 was decreased in aged mouse muscle; thus, our data reproduce previous findings.

The processing of pre-miRNA produces a miRNA duplex consisting of an miRNA (guide) and miRNA* (passenger) strand [36]. Although the miRNA* strand is generally considered to have no functional role due to its low abundance, some miRNA* have been detected with high abundance during large-scale small RNA sequencing. Furthermore, some miRNA* strands were shown to be incorporated into RISC-complex and to show inhibitory activity, suggesting the impact of miRNA* species on regulatory networks [37]. Of note, in our dataset, five miRNAs, miR-136, -337, -434, -3071, -3544, exhibited a high dominance in both the guide and passenger strands. These five miRNA/miRNA*s were preferentially down-regulated with aging (Table 2). It remains unknown whether the down-regulated miRNA* strands, miR-136-3p, -337-3p, -434-5p, -3071-3p, and -3544-5p, have regulatory roles contributing to muscle aging.

Most mammalian miRNA genes are scattered individually in diverse genomic locations, but some miRNA genes are clustered in the genome [38]. There are miRNA clusters that contain two to three miRNA genes or mega-clusters that contain >50 miRNA genes. The clustered miRNAs are often oriented in the same direction and transcribed in a polycistronic manner with similar expression patterns. The imprinted *Dlk1-Dio3* genomic region, which is located on human chromosome 14 (mouse chromosome 12), contains the paternally expressed genes *Dlk1, Rtl1*, and *Dio3* and the maternally expressed non-coding RNA genes *Meg3* (*Gtl2*) and *Meg8* (*Rian*) as well as the antisense *Rtl1* (*anti-Rtl1*) [39]. Furthermore, the *Dlk1-Dio3* region contains 54 miRNAs, the largest miRNA cluster found in the human genome [40]. Notably, 8 of the 14 down-regulated miRNAs (57%) identified in this work belonged to the miRNA cluster derived from the *Dlk1-Dio3* region on mouse distal chromosome 12 and changed in the same direction in muscle. The miRNA clusters within the *Dlk1-Dio3* region are organized in two groups; the smaller one between *Gtl2* and *Rian* and the larger one between *Rian* and *Mirg* (also known as the miR-379-410 cluster); seven miRNAs found in skeletal muscle were derived from the smaller cluster, *Gtl2/Rian* (Fig. S3).

The miRNA clusters and genes within the *Dlk1-Dio3* region are differentially regulated in various diseases such as cancer and schizophrenia as well as skeletal muscle development [41]. The expression of ovine *Dlk1, Gtl2, Rtl1*, and *Meg8* is enriched in skeletal muscle, and overexpression of *Dlk1* and *Rtl1* is strongly correlated to muscle hypertrophy in *Callipyge* sheep [42]. The *Gtl2-Dio3* miRNA cluster-mediated regulation of WNT signaling by MEF2A is required for skeletal muscle regeneration [43]. Of the eight miRNAs located in the imprinted *Dlk1-Dio3* region, four miRNAs, miR-127, -136, -434, and -3071, are located within *anti-Rtl1*, which contains seven miRNAs (Fig. S3, based on miRbase). In this respect, these observations raise the questions of (1) if the miRNA cluster within the *Dlk1-Dio3* region is involved in muscle aging by participating in muscle regeneration or muscle atrophy; and (2) whether silencing of the miRNA cluster within the *Dlk1-Dio3* region with aging occurs in other tissues. By the integrated analysis of small RNA-Seq and mRNA-Seq data, we identified 94 mRNAs targeted by 14 age-regulated miRNAs using the TargetScan algorithm. Interestingly, GO functional clustering analyses revealed that genes targeted by down-regulated miRNAs, such as the Ccna2, Egr2, Klf4, Klf6, Mafb, Nfya, and Rybp genes, are mainly involved in the regulation of transcription, whereas genes targeted by up-regulated miRNAs are not enriched in any of the GO categories. Recently, it was reported that Ring1 and YY1-binding protein (Rybp) is down-regulated by miR-29 during myogenesis and acts as a negative regulator of skeletal myogenesis in injury-induced muscle regeneration [32]. These results suggested that Rybp and its putative regulatory miRNA, miR-136, could contribute to muscle aging process. Future investigations into precise regulatory mechanisms for Rybp and miR-136 are needed.

In summary, we have identified 34 age-related miRNAs in the skeletal muscle, and of those 34, over 50% of down-regulated miRNAs are enriched as a cluster in the *Dlk1-Dio3* imprinted genomic region. By analyzing miRNA-mRNA interactions, we have identified that the miRNA contributes to muscle aging primarily through the regulation of transcription. Therefore, our results provide a clue regarding the role of miRNA in skeletal muscle aging.

## METHODS

### Sample collection

Six young (6-month-old) and six aged (24-month-old) male C57BL/6 mice were purchased from the Laboratory Animal Resource Center (Korea Research Institute of Bioscience and Biotechnology). The body weight of the mice was recorded before sacrifice for the muscle dissection into four types of muscles, soleus (SOL), extensor digitorum longus (EDL), tibialis anterior (TA), and gastrocnemius (*n* = 12). Each muscle mass was recorded, and the gastrocnemius muscle was immediately selected for further experiments.

### MiRNA sequencing and Data analysis

Small RNA-enriched total RNA from the gastrocnemius muscle (*n* = 12) was extracted using the *mir*Vana miRNA isolation kit (Ambion, Austin, TX) according to the manufacturer's protocol, and miRNA libraries were prepared following the Illumina library preparation protocol (Illumina Inc., San Diego, CA, USA). Each library was indexed with the Illumina adaptor (6-base barcode). The small RNA library was size fractionated on a 6% TBE urea polyacrylamide gel, and the 140-160 base pair fraction was excised from the gel. The purified miRNA library was quantified on the Agilent DNA 1000 chip. All indexed samples were pooled and sequenced in one lane of Illumina GAIIx (36 bp reads). Before mapping the raw small RNA sequencing reads on the reference genome, the base qualities of the reads were evaluated by FastQC (http://www.bioinformaticsbabraham.ac.uk/projects/fastqc), and the attached adaptor sequences at the 3' ends were removed by an in-house PERL script. After trimming the adapter sequences, the read size distribution was analyzed by an R (http://www.r-project.org.) script. Adapter-trimmed small RNA reads were then mapped on the reference genome mm10 downloaded from the UCSC Genome Browser (http://genome.ucsc.edu) by the BWA (http://bio-bwa.sourceforge.net.) software with the default options [44]. To assign the RNA classes for the mapped reads, we used the genomic position information of small RNAs and repeats in the annotation files from the UCSC Genome Browser and fRNAdb (http://www.ncrna.org/frnadb.) for miRNAs, tRNAs, rRNAs, and other non-coding RNAs [45]. By comparing the mapping position and annotation information, all mapped reads were classified into the corresponding RNA types, and the relative abundance of expression of each RNA type was evaluated based on the assigned read counts. To determine the portion of the main miRNA expression in the total small RNA sequencing data, we selected the top 10 expressed miRNAs from the pooled read set of 6 young mouse samples and evaluated the relative portion of the reads for those 10 most abundant miRNAs and other small portioned reads in the pooled young and aged mouse read sets.

For the known miRNAs (miRBase ver. 20), we calculated fragments per kilobase of transcript per million fragments mapped (FPKM) for each precursor and mature miRNA from the mapped reads using a custom Python script. For novel miRNA discovery, we used the mirDeep2 software (version 2.0.5) with the default parameters. MiRBase was used to filter known miRNAs, and *Rattus norvegicus* was used as a related species.

### RNA sequencing and data analysis

Total RNA was extracted from young and aged gastrocnemius muscle (*n* = 5 for each group) using the RNeasy Fibrous Tissue Mini Kit according to the manufacturer's instructions (Qiagen). The quality and integrity of the RNA were confirmed by agarose gel electrophoresis and ethidium bromide staining, followed by visual examination under ultraviolet light. The sequencing library was prepared using the TruSeq RNA Sample Preparation kit v2 (Illumina, CA, USA) according to the manufacturer's protocols. In brief, mRNA was purified from the total RNA using poly-T oligo-attached magnetic beads, fragmented, and converted into cDNA. Adapters were then ligated, and the fragments were amplified by PCR. The sequencing was performed in paired end reads (2×76 bp) using a Genome Analyzer IIx (Illumina).

Reference genome sequence data from *Mus musculus* were obtained from the University of California Santa Cruz Genome Browser Gateway (assembly ID: mm10). The reference genome index was built using the Bowtie2-build component of Bowtie2 (ver. 2.0) and SAMtools (ver. 0.1.18). Tophat2 (version 2.0.8) was applied to tissue samples to map the reads to the reference genome. Gene expression was measured as FPKM for each gene using the Refseq gene (mm10) model downloaded from the UCSC Browser gateway. Each FPKM was log_2_-transformed for further analysis.

### Mature miRNA expression analyses

TaqMan MicroRNA Assays were performed according to the manufacturer's recommended protocols (Applied Biosystems). Briefly, each reverse transcriptase (RT) reaction contained 10 ng of total RNA isolated using the *mir*Vana miRNA isolation kit, 50 nM stem-loop RT primer, 0.25 mM each dNTP, 3.33 units/µl MultiScribe RT enzyme, 0.25 units/µl RNase inhibitor, and 1 × RT buffer (all purchased from Applied Biosystems). The reactions were incubated for 30 min at 16 °C, 30 min at 42 °C, and 5 min at 85 °C and then held at 4°C. Each quantitative PCR (qPCR) reaction included 1.33 µl of RT product, 1 µl of the 20 × primers and probe mix of the TaqMan MicroRNA Assay, 0.5 µl of H-Taq DNA polymerase (SolGent, Daejun, Korea), and 10 µl of homemade buffer mixture [40 mM Tris-HCl (pH8.4), 100 mM KCl, 6 mM MgCl_2_, 0.5 mM each dNTP, and 10% DMSO] instead of the 2 × TaqMan Universal PCR Master Mix in a 20-µl reaction volume. The reactions were incubated in a 96-well plate on a Bio-Rad CFX96 system at 95°C for 10 min, followed by 40 cycles of 95°C for 15 s and 60°C for 1 min. The threshold cycle (Ct) is defined as the fractional cycle number at which the fluorescence passes the fixed threshold. U6 snRNA served as an endogenous control for normalization.

### Gene functional analysis

To characterize the biological processes affected by age-related mRNAs and miRNA, we used Gene Ontology (GO) and functional annotation clustering to classify the similar annotations into a cluster of the Database for Annotation, Visualization and Integrated Discovery (DAVID).

### Prediction of miRNA-mRNA interactions and network construction

We used the TargetScan (version 6.2, http://www.targetscan.org) database to predict potential target mRNAs for each miRNA. Based on the predicted miRNA-mRNA relationships, we selected miRNA-mRNA pairs showing opposite expression changes. The log_2_-fold change over 0.5 was used as a cutoff. The network between miRNAs and mRNAs was created using Cytoscape (version 3.0, www.cytoscape.org/).

### Quantitative real time RT-PCR

Total RNA was isolated using Qiazol reagent (Qiagen, Valencia, CA) followed by quantitative real time PCR using the StepOnePlus real-time PCR system (Applied Biosystems). Real-time PCR reactions were performed using specific primers at 95 °C for 3 min, followed by 40 cycles of 95 °C for 20 s, 60 °C for 20 s, and 72 °C for 20 s. The GAPDH housekeeping gene served as an endogenous control for normalization. The following primers were used: Egr2 forward 5'-TCAGTGGTTTTATGCACCAGC-3', Egr2 reverse 5'-GAAGCTACTCGGATACGGGAG-3', Klf4 forward 5'-GTGCCCCGACTAACCGTTG-3', Klf4 reverse 5'-GTCGTTGAACTCCTCGGTCT-3', Klf6 forward 5'-GTTTCTGCTCGGACTCCTGAT-3', Klf6 reverse 5'-TTCCTGGAAGATGCTACACATTG-3', Rybp forward 5'-AGGCCAAAAAGACAAGCGAAA-3', Rybp reverse 5'-TGAGAATTGATGCGAGGTTTCC-3'.

### Western blot analysis

The following antibodies were used in this study according to standard protocol: anti-GAPDH (Santa cruz) and anti-Rybp (Millipore).

### Statistical analysis

The data were analyzed with Microsoft Excel using Student's t-test, and probability values of less than 0.05 were considered significant. The data are presented as the mean, and the error bars indicate the standard error of the mean (SEM).

### Data deposition

The miRNA sequencing data are available at the National Center for Biotechnology Information (NCBI) Gene Expression Omnibus (GEO) and are accessible through GEO Series accession number GSE55164. The RNA sequencing data are accessible through GEO accession number GSE55163.

## SUPPLEMENTAL DATA


