# Supplementary material for: Genome-wide profiling of the microRNA-mRNA regulatory network in skeletal muscle with aging
Source: Aging (Albany NY). 2014 Jul 12;6(7):524–44. doi: 10.18632/aging.100677 (PMC4153621; doi:10.18632/aging.100677)
Supplement: Supplementary file 1 [file aging-06-524-s001.pdf]

SUPPLEMENTAL DATA

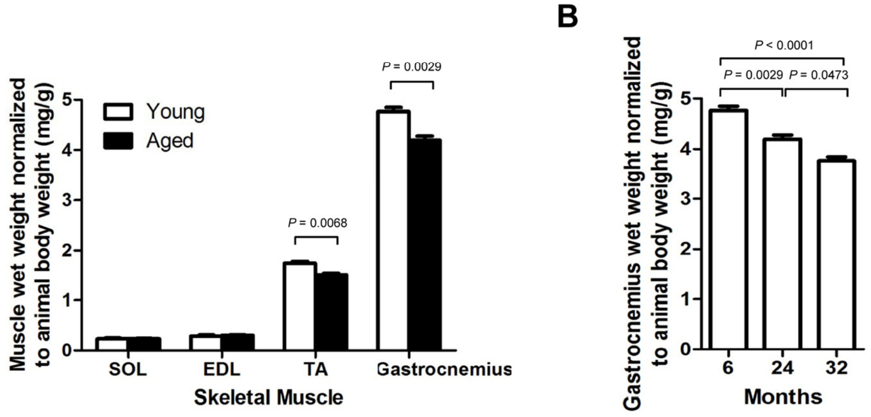

Figure S1. Muscle mass changes in different anatomical regions with age. (**A**) The TA and gastrocnemius muscle showed a significant loss of weight in aged mice (24-month-old) compared to young mice (6-month-old) (*n* = 12 for each group). Muscle weight was normalized to animal body weight. Soleus, SOL. Extensor digitorum longus, EDL. Tibialis anterior, TA. (**B**) The mass of the gastrocnemius muscle gradually decreased through 32 months of age.

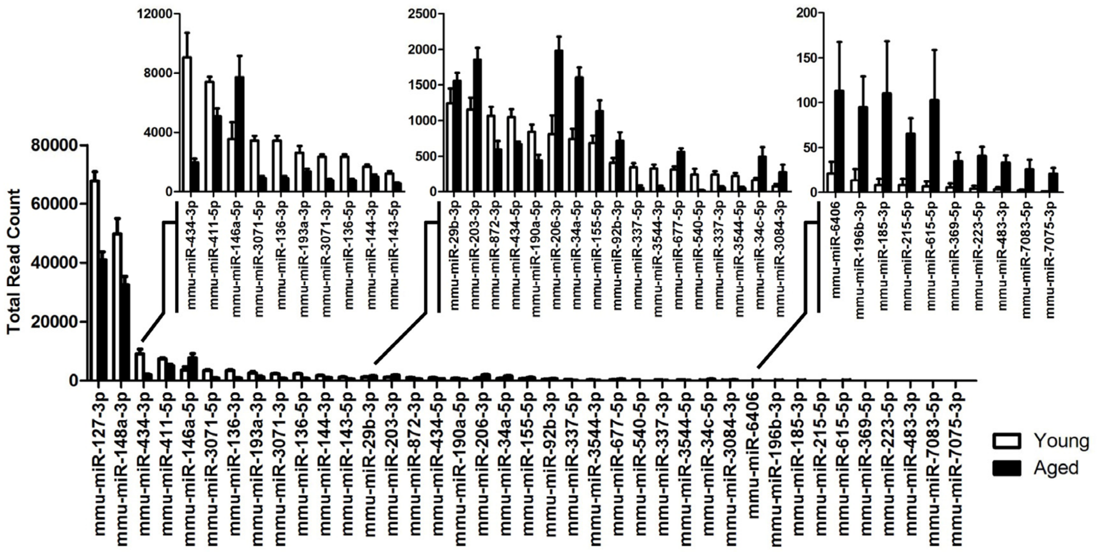

Figure S2. Total read count for 39 differentially expressed miRNAs with aging in skeletal muscle. Inset graph shows a magnified value for low read counts. Data are presented as the mean ± SEM. White bar; young muscle, black bar; aged muscle.

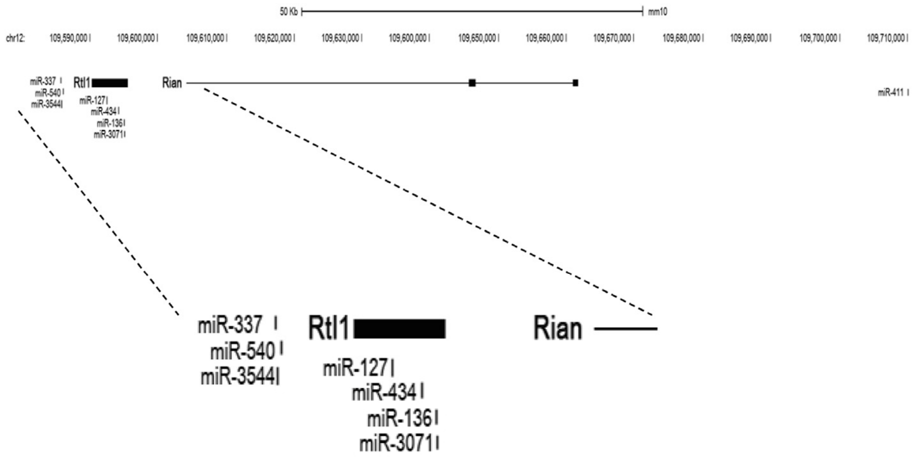

Figure S3. Eight down-regulated miRNAs located in *Dlk1-Dio3* genomic regions. Genomic browser image around *Rtl1 and Rian*. Top, positions of eight down-regulated miRNAs are shown with the chromosomal location marked at the top. Bottom, seven miRNAs are distributed near the *Rtl1* as indicated in the magnified view.

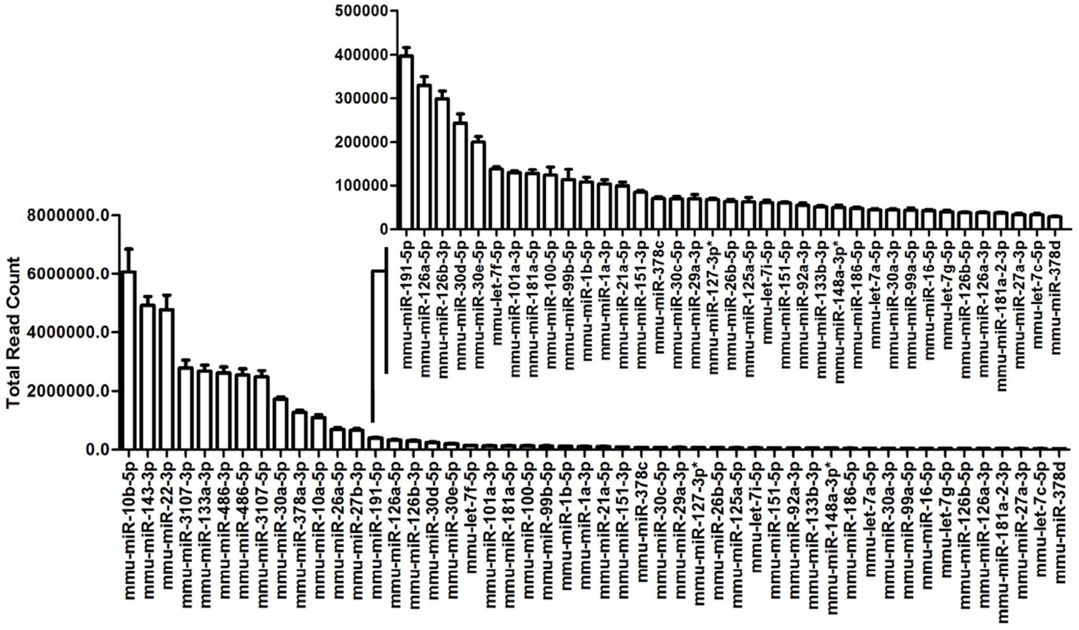

Figure S4. Fifty most abundant miRNAs identified in skeletal muscle. Data are presented as the normalized total read counts.

Table S1. 16 novel miRNAs identified in skeletal muscle. The following information is presented from left to right: provisional miRNA ID, genomic coordinate and strand (+ or -), mature sequence, miRNA length, genomic location, and mature/star read counts.

Provisional ID	Genomic location	Mature sequence	Length	Location	Mature read	Star read
chr7_8267	chr7:16471899..16471933:+	ACCGGGUGCUGUAGGCUU	18	Intronic	924	0
chr13_816	chr13:84058941..84058983:+	UGAGAUGAAACACUGUAGCA	20	Exonic	22	0
chr2_11277	chr2:166576728..166576783:-	CCGGGUGCUGUAGGCACU	18	Intronic	38	0
chr2_5897	chr2:165234414..165234461:-	GGCGCGGCGCGGGCUCCG	18	Intronic	26	0
chr7_7384	chr7:121040347..121040416:-	UGAUUGGAAGACACUCUGCAAU	22	Intronic	20	0
chr11_2952	chr11:87448818..87448900:+	GGGAGGGAACGCAGUCUGAGUGG	23	Intronic	44	0
chr10_1092	chr10:130557384..130557462:	GAGAGGAACAACUCUGAGUCU	21	Intergenic	19	0
chr12_1908	chr12:33262824..33262884:+	UCAGAACAACCUGACCUGCCU	21	Intronic	23	1
chr3_5770	chr3:147057717..147057785:-	CACCAGGAGUGGAGCCUGC	19	Intergenic	11	0
chr4_5989	chr4:155858806..155858863:+	UUCAAACCUCUCUGGCUGCC	20	Exonic	8	0
chr1_524	chr1:133827269..133827312:-	UGAGAUGAAGCCCUGUAGG	19	Intergenic	6	0
chr19_14188	chr19:5840778..5840840:-	CGGGGUGAUCGGAUGGCCG	19	Intergenic	2	0
chr8_7686	chr8:21095625..21095687:-	GAUAAAUGGAGUCACAGACAU	21	Intronic	13	0
chr11_3878	chr11:120633334..120633383:-	CGGGGCUGGGGCGGGCGG	18	Intronic	1	0
chr18_4213	chr18:34759521..34759596:+	CCCAUGGAGCUGUAGGAGCCG	21	Intronic	16	0
chr2_4292	chr2:28495933..28495984:+	AUCUCGCUGGGGCCUCCA	18	Intergenic	57	0

Table S2. Functional annotation clustering of enriched GO terms stimulated by down-regulated miRNAs in aged muscle. The up-regulated genes targeted by down-regulated miRNAs were subjected to gene ontology analysis with DAVID functional annotation clustering. The genes targeted by down-regulated miRNAs represented two clusters with enrichment scores ≥1.3. ( )*; an enrichment score of 0.05 is equivalent to an enrichment score of 1.3 in the minus log scale [1].

**Annotation cluster**	**Category**	**Term**	**Count**	***P*-value**
Cluster 1 (1.5)*	GO cellular component	GO:0044432~endoplasmic reticulum part	4	0.006473
		GO:0005783~endoplasmic reticulum	5	0.048089
Cluster 2 (1.3)*	GO biological process	GO:0045941~positive regulation of transcription	6	0.001521
		GO:0010628~positive regulation of gene expression	6	0.001714
		GO:0045935~positive regulation of nucleobase, nucleoside, nucleotide and nucleic acid metabolic process	6	0.002081
		GO:0051173~positive regulation of nitrogen compound metabolic process	6	0.002382
		GO:0010557~positive regulation of macromolecule biosynthetic process	6	0.002462
		GO:0031328~positive regulation of cellular biosynthetic process	6	0.002938
		GO:0009891~positive regulation of biosynthetic process	6	0.003054
		GO:0045944~positive regulation of transcription from RNA polymerase II promoter	5	0.003867
		GO:0006357~regulation of transcription from RNA polymerase II promoter	6	0.004703
		GO:0010604~positive regulation of macromolecule metabolic process	6	0.005279
		GO:0045893~positive regulation of transcription, DNA-dependent	5	0.006577
		GO:0051254~positive regulation of RNA metabolic process	5	0.006744
	GO molecular function	GO:0030528~transcription regulator activity	7	0.011563
		GO:0003700~transcription factor activity	5	0.036412

Table S3. Human homologues for 3 down-regulated miRNAs are related to muscular disease. This table shows three human homologues for down-regulated miRNAs identified in aged skeletal muscle and their previously reported expression in each muscular disease. The three miRNAs are related to 6 muscular diseases.

Muscular disease	miRNAs	Expression
Dermatomyositis (DM)	hsa-miR-148a	up-regulated
Duchenne muscular dystrophy (DMD)	hsa-miR-148a	up-regulated
Miyoshi myopathy (MM)	hsa-miR-148a	up-regulated
Limb-girdle muscular dystrophies types 2A (LGMD2A)	hsa-miR-148a	up-regulated
Nemaline myopathy (NM)	hsa-miR-127	up-regulated
Polymyositis (PM)	hsa-miR-127	up-regulated

Table S4. Human homologues for 5 up-regulated miRNAs are related to muscular disease. This table shows five human homologues for up-regulated miRNAs identified in aged skeletal muscle and their previously reported expression in each muscular disease. The five miRNAs are related to 10 muscular diseases.

Muscular disease	miRNAs	Expression
Becker muscular dystrophy (BMD)	hsa-miR-146b	up-regulated
Dermatomyositis (DM)	hsa-miR-223	up-regulated
Duchenne muscular dystrophy (DMD)	hsa-miR-369-5p	up-regulated
Facioscapulohumeral muscular dystrophy (FSHD)	hsa-miR-369-5p	up-regulated
Inclusion body myositis (IBM)	hsa-miR-223	up-regulated
Limb-girdle muscular dystrophies types 2A (LGMD2A)	hsa-miR-223	up-regulated
Miyoshi myopathy (MM)	hsa-miR-223	up-regulated
Nemaline myopathy (NM)	hsa-miR-223	up-regulated
Polymyositis (PM)	hsa-miR-34a	up-regulated
Rhabdomyosarcoma (RMS)	hsa-miR-29b-2	down-regulated

REFERENCES

1. Huang da W, Sherman BT, Tan Q, Collins JR, Alvord WG, Roayaei J, Stephens R, Baseler MW, Lane HC and Lempicki RA. The DAVID Gene Functional Classification Tool: a novel biological module-centric algorithm to functionally analyze large gene lists. Genome biology. 2007; 8:R183.
